# Supplementary material for: Complexome profiling of the Chlamydomonas psb28 mutant reveals TEF5 as an early PSII assembly factor
Source: Plant Cell. 2025 Mar 17;37(6):koaf055. doi: 10.1093/plcell/koaf055 (PMC12164590; doi:10.1093/plcell/koaf055)
Supplement: koaf055_Supplementary_Data [file koaf055_supplementary_data.zip › psb28_interactive.zip!/plots/intensity/qualityPlots/peptides/AGT1_Cre06.g294650.t1.2.html]

### Cre06.g294650.t1.2

Trivial name: AGT1  
SeqCoverage [median]: 53.80  
Mass: 46.06 kDa  
  
 

| Sequence | MissedCleavage | Mass [Da] | wtIntensity | muIntensity | Score |
| --- | --- | --- | --- | --- | --- |
| AALTQHKPK | 1 | 992.58 | 920.74 | 0.00 | 83.27 |
| AMEALQK | 0 | 789.41 | 21147.69 | 20176.84 | 154.92 |
| ASEEVVAVDNAAR | 0 | 1329.65 | 35418.38 | 46037.31 | 243.53 |
| ASEEVVAVDNAARK | 1 | 1457.75 | 0.00 | 665.90 | 62.89 |
| EALAIVSEEGLEK | 0 | 1386.72 | 23587.61 | 49040.24 | 177.47 |
| HAMDTYSLEIAGGLGPTAGK | 0 | 1987.97 | 14734.23 | 16830.82 | 131.11 |
| ILTAQALPLLGHMHPPFLK | 0 | 2096.20 | 17611.92 | 9786.71 | 77.75 |
| IMDELSEGLR | 0 | 1161.57 | 18981.82 | 29999.47 | 187.82 |
| LITVNTIK | 0 | 900.56 | 2249.41 | 6307.17 | 113.89 |
| LLMGPGPANAYPR | 0 | 1355.70 | 30446.89 | 43520.51 | 193.38 |
| LWDGLR | 0 | 758.41 | 0.00 | 1944.68 | 74.83 |
| RYNADVIELK | 1 | 1219.66 | 1183.94 | 6937.62 | 105.52 |
| SMGLKPFVEKDEDR | 2 | 1649.81 | 2872.99 | 5471.09 | 67.27 |
| SYHHTGPVSTFYAMR | 0 | 1752.80 | 9225.10 | 9026.69 | 117.03 |
| TPAGTTFSYETLK | 0 | 1414.70 | 30087.77 | 60062.11 | 291.24 |
| VGIMGFNAKPQNVDLVLTAFQDGLKR | 2 | 2830.52 | 5374.88 | 0.00 | 96.90 |
| VLVGNAGIWGER | 0 | 1269.68 | 4931.40 | 4874.97 | 102.06 |
| VPEGVDWAAVVK | 0 | 1268.68 | 1776.21 | 6397.74 | 114.40 |
| YNADVIELK | 0 | 1063.56 | 9824.23 | 9412.81 | 137.14 |
| YTFQTK | 0 | 786.39 | 0.00 | 13896.44 | 142.63 |
|  |  |  | 230375.2 | 340389.1 |  |
